# Cherenkov light emission in external beam radiation therapy of the larynx

**DOI:** 10.1117/1.JBO.30.5.055002

**Published:** 2025-05-29

**Authors:** Jigar Dubal, Pedro Arce, Chris South, Lucia Florescu

**Affiliations:** a University of Surrey, Centre for Vision, Speech and Signal Processing, United Kingdom; b CIEMAT (Centro de Investigaciones Energéticas, Medioambientales y Tecnológicas), Madrid, Spain; c Royal Surrey County Hospital NHS Foundation Trust, Department of Medical Physics, Guildford, United Kingdom

**Keywords:** Cherenkov light, radiation therapy, spatial and spectral characteristics

## Abstract

**Significance:**

Cherenkov light emitted in the tissue during radiation therapy enables unprecedented approaches to tumor functional imaging for early treatment assessment. Cherenkov light–based tomographic imaging requires image reconstruction algorithms based on internal light sources that, in turn, require knowledge about the characteristics of the Cherenkov light within the patient.

**Aim:**

We aim to investigate the spatial and spectral characteristics of Cherenkov light within the patient and at the patient’s surface, and the origin within the tissue of light reaching the surface, to provide insight for the development of image reconstruction algorithms for Cherenkov light–based tomographic imaging.

**Approach:**

Numerical experiments using clinical patient data and Monte Carlo simulations are performed for the radiation therapy of laryngeal cancer for intensity-modulated radiation therapy and volumetric-modulated arc radiation therapy.

**Results:**

The emitted Cherenkov light is concentrated in regions of high delivered dose, with the spatial distribution within the patient and at the patient’s surface depending on the treatment type and patient anatomy. The Cherenkov light at the patient’s surface is dominant in the near-infrared spectral region. Light emitted within the tumor emerges at the patient’s surface on a well-defined radiation beam–independent region. The distribution within the patient of the emitted light that emerges on reduced areas on the patient’s surface containing this region is similar to that of the light that emerges across the entire patient’s surface.

**Conclusions:**

Detailed information about the spectral and spatial characteristics of Cherenkov light is provided. In addition, these results suggest that surface light measurements restricted to smaller areas containing the region where the light emitted in the tumor emerges (that can be determined through simulations prior to the treatment) could enable probing the tumor while being easier to integrate with the radiotherapy system and while the effect of measurement data incompleteness on image reconstruction may not be too strong.

## 1 Introduction

External beam photon radiation therapy (EBRT) utilizes high-energy X-ray photon beams produced by a linear accelerator to deliver radiation doses to the diseased tissue. As the X-ray photons interact with biological tissue, charged particles are produced, which propagate and deposit energy as radiation dose along the way. In addition to depositing dose, the charged particles that have a high enough energy and a phase velocity exceeding the phase velocity of light in the tissue also induce the emission of Cherenkov light.^1^ The ability to measure at the patient’s surface Cherenkov light produced during radiation therapy^2^^–^^4^ opens new avenues for applications to radiation therapy dosimetry^4^^,^^5^ and tissue functional imaging.^3^^,^^6^^,^^7^

The outcome of radiation therapy depends on the ability to assess the treatment efficacy early into the several-week-long treatment course and adapt the treatment accordingly. In this context, monitoring tumor metabolism and hemodynamics plays an important role, as these are early indicators of treatment efficacy and predictors for tumor response to treatment.^8^ Optical imaging^9^ has unique potential for this purpose while also being non-ionizing and cost-effective. Indeed, near-infrared diffuse optical spectroscopy and imaging with external light sources have been demonstrated to enable early assessment of tumor response to therapy, with changes in tumor characteristics observed in as little as a few hours after the first session of treatment.^10^^–^^13^ Furthermore, tumors may experience intermittent blood flow resulting in transiently hypoxic cells,^14^ and the dynamics of tumor oxygenation during treatment delivery has also been identified as a marker for treatment efficacy.^15^^,^^16^ Therefore, monitoring the tumor hemodynamics during radiation delivery can provide a comprehensive treatment assessment.

Recently, optical spectroscopy and imaging using Cherenkov light have been considered for tumor assessment during radiation therapy^6^^,^^7^^,^^17^ in phantom and small-animal studies. The potential of Cherenkov light emitted in tissue during treatment to excite exogenous phosphorescent agents to probe tissue oxygenation^7^^,^^18^^–^^21^ has also been demonstrated. The advantage of using Cherenkov light, rather than external light sources, for tissue imaging, is due to the fact that it is inherently produced in the treated tissue, thus providing access to the whole volume of interest. In addition, as the distribution of the treatment radiation is accurately known, Cherenkov light can be spatially well-characterized, enabling optimized detection. In addition, as it does not require the hardware associated with the light sources, Cherenkov light–based imaging can be implemented more easily with the radiation therapy technology for *in vivo*, real-time tumor imaging during treatment delivery. Tissue tomographic imaging using Cherenkov light, however, requires novel image reconstruction algorithms based on internal (rather than external) light sources, and knowledge about the distribution in the tissue of the emitted Cherenkov light (that represents the internal light sources) is needed to guide the development of formalisms for such reconstruction algorithms.

This study investigates the characteristics of Cherenkov light emission within a patient in photon EBRT, with the aim to provide insight into the development of Cherenkov light–based tomographic imaging techniques, including techniques not requiring exogenous contrast agents, for tumor assessment during radiation therapy. Specifically, we investigate Cherenkov light emission during EBRT of laryngeal cancer, an anatomical site for which optical tomography may be feasible. We consider intensity-modulated radiation therapy (IMRT) and volumetric-modulated arc therapy (VMAT), the two main EBRT treatment modalities. In IMRT treatments, a small number of crossing radiation beams of specific shape and intensity are delivered (at different times) from fixed angular positions of the rotating gantry (source of radiation), whereas in VMAT treatments, radiation is delivered continuously as the gantry rotates around the patient along a certain arc, with the radiation beam shape and intensity modulated as a function of gantry position. Although the dose delivered to the diseased tissue is the same in both treatments, due to the difference in radiation delivery, there is a difference between these treatment types in the spatial distribution of dose and Cherenkov light in the surrounding healthy tissue and within the body. Characterizing and illustrating Cherenkov light emission in each case could provide insight for developing (possibly treatment delivery-specific) formalisms and approaches to image reconstruction. For both treatment types, we perform numerical experiments using clinical patient data to investigate the spectral and spatial characteristics of Cherenkov light emitted within the patient and emerging at the patient’s surface, as well as to determine the origin of light within the patient emerging on certain regions on the patient’s surface, to provide insight for exploiting such characteristics to probe the tumor. In particular, motivated by the aim to reduce the measurement area while ensuring that the diseased tissue is still accurately probed, we illustrate how to identify regions on the patient’s surface where light emitted in the tumor emerges, compute the surface light intensity (photons per unit area), and provide insight for the level of data incompleteness that need to be considered and corrected for when deriving image reconstruction algorithms based on such reduced area measurements. The characteristics of Cherenkov light within tissue and the origin within the tissue of light emerging on the patient’s surface can only be investigated by performing numerical (rather than physical) experiments such as those in this study.

## 2 Numerical Experiments

In this study, we use Monte Carlo simulations and clinical radiotherapy treatment data to model the transport of the treatment radiation, dose deposition, and Cherenkov light emission and propagation within the patient. Monte Carlo simulations account for all relevant processes with high accuracy. Simulations are performed using the Geant4 toolkit—GAMOS (Geant4-based Architecture for Medicine-Oriented Simulations)^22^ and the GAMOS tissue optics plug-in.^23^ Clinical computed tomography (CT) data are utilized to define the sample (patient) geometry and characteristics, and the corresponding patient structure data (RTStruct) provides segmentation of relevant tissue types. The treatment planning data (RTPlan) is utilized in conjunction with the linear accelerator phase space data to simulate the patient- and treatment type–specific radiation delivery.

### 2.1 Patient Geometry

The IMRT and VMAT treatments considered in this study are for different patients. For each patient, a voxelized patient geometry is created based on 3D CT data, with a voxel size of 1.0  mm×1.0  mm×1.3  mm. Using the CT scanner–specific calibration curve, the values of each voxel in the CT image are converted to a material mass density, which is then utilized to define the tissue type. The following tissue and material types were used: skin, adipose, muscle, bone, tumor (represented in GAMOS by soft tissue), and air.

**Fig. 1:**
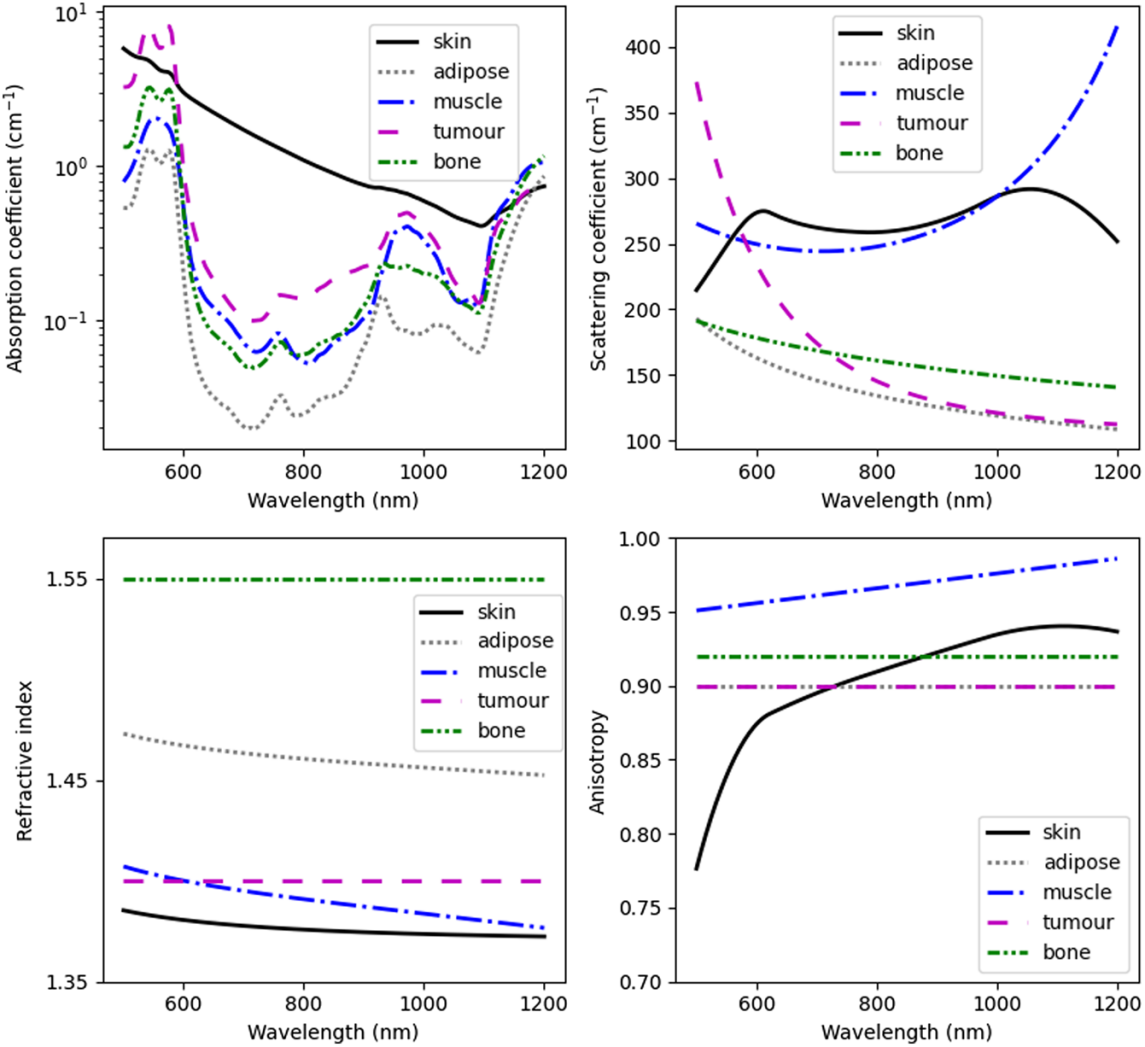
Optical properties of the biological tissue used in this study. The graphs for tissue anisotropy factor for tumor and adipose tissue overlap.

Each voxel was assigned by GAMOS tissue-specific X-ray characteristics (such as the linear attenuation coefficient and ionization energy) obtained from the National Institute of Standards and Technology database.^24^ Furthermore, each tissue type was assigned spectrally dependent optical absorption and scattering coefficients, anisotropy, and refractive index. The spectral range used in this study is 500 to 1200 nm, covering the broad Cherenkov light emission spectrum and including the near-infrared region, in which light absorption in biological tissue is minimal. The wavelength range below 500 nm was not included as light absorption by the tissue in this spectral range is large, and light emitted deeper in the tissue and in the tumor does not contribute to surface light.^25^ The optical parameters of skin, adipose, muscle, and bone tissue were determined as given in Refs. 5, 25, and 26. The optical scattering and absorption coefficients of the tumor were computed as given in Ref. 5 using available experimentally measured parameters for a fibrous tumor^25^ and head-and-neck tumors,^26^ respectively; its anisotropy factor was considered the average anisotropy factor of biological tissue,^27^ and its refractive index the same as for an esophagus tumor.^28^ The optical characteristics used in this study are presented in Fig. 1.

### 2.2 Treatment Delivery

**Fig. 2:**
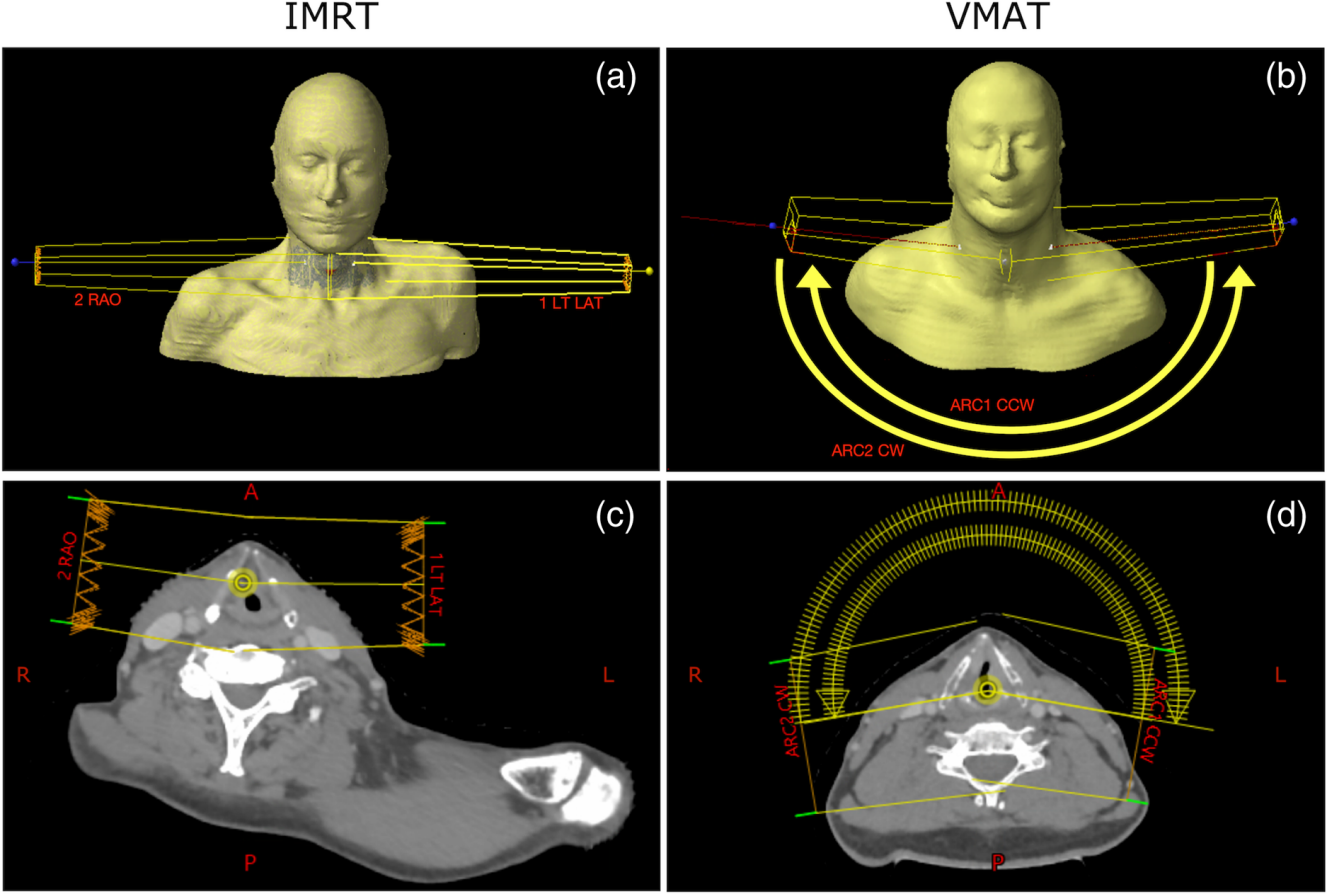
3D view [(a) and (b)] and 2D view at an axial slice through the isocenter [(c) and (d)] of the two treatment beams for the IMRT treatment [(a) and (c)] and the two treatment arcs and radiation beams at the start of the arcs for the VMAT treatment [(b) and (d)]. L, R, A, and P indicate left, right, anterior, and posterior positions, respectively.

The treatments considered in this study were performed using a Varian TrueBeam linear accelerator. To simulate the treatment delivery, phase space data provided by the manufacturer are used as the radiation source. These data provide information about the energy, position, and direction of X-ray photons that form the radiation beam produced by the linear accelerator, at a position above the beam-shaping components—the jaws and multileaf collimators (MLCs). The beam-shaping components utilized clinically were modeled in GAMOS using their design specifications. For the IMRT and VMAT treatments considered here, the MLCs used are 120MLC and HDMLCs, respectively. The RTPlan data provide the information for patient- and treatment-specific modeling of the radiation beam for each gantry angle, in particular, to simulate the position and geometry of the beam-shaping components. The Varian TrueBeam linear accelerator is modeled in GAMOS with a dose rate of $600\;{\mathrm{cGys}}^{-1}$ The IMRT treatment considered in this study is delivered via two radiation beams delivered at two gantry angles (defined as the angle between the direction of the radiation beam and the direction of gravity). The VMAT treatment consists of two arcs of radiation delivery of 200 deg each. For both treatments, the nominal radiation energy is 6 MV, and the total dose was delivered through 20 identical daily treatment sessions (fractions). Other relevant treatment characteristics are presented in Table 1, and Fig. 2 illustrates the treatment beams and arcs for IMRT and VMAT treatments, respectively, where the realistic 3D representation of the patient geometry was obtained from the CT data, and the 2D slice is an axial slice through the isocenter (the point of intersection of the axes of rotation of various linear accelerator components, which is positioned in close proximity to the center of the tumor for the treatments considered in this study). The Monte Carlo simulation of both treatments was carried out using $4.7\times 10^{8}$ particles from the phase space files (events).

**Table 1:** Beam- and arc- specific treatment characteristics for the IMRT and VMAT treatments, respectively, indicating the number of control points (CP), the monitor units (MUs), and the gantry angle (GA) value (for IMRT) and value range (for VMAT) for radiation delivery. The IMRT beams are a right anterior oblique (RAO) beam and a left lateral (LT LAT) beam. For VMAT, the arcs of radiation 1 and 2 are delivered as the gantry rotates counter-clockwise (CCW) and clockwise (CW), respectively.

Treatment	Leaf-type	Beam/arc	CPs	MUs	GA (°)
IMRT	120MLC	1, (LT LAT)	8	157.5	90
		2, (RAO)	10	155.1	278
VMAT	HDMLC	1, (CCW)	114	360.7	100 to 260
		2, (CW)	114	306.6	260 to 100

### 2.3 Dose and Cherenkov Light Computation

The dominant interaction between the therapeutic X-ray photons and biological tissue is Compton scattering.^29^ As a result of this interaction, charged particles are produced in the tissue that are responsible for dose delivery. The voxelized absorbed dose in units of gray (Gy) per simulated Monte Carlo event (particle) is calculated as the total energy deposited in the voxel divided by the mass of the voxel (determined from its volume and density provided by CT data). The simulated dose was converted to Gy by assigning a known dose value for a known amount of radiation to a given point. The clinical calibration of the linear accelerator ensures^30^ that 1 MU of radiation delivers a dose of 1 cGy at a depth d=1.5  cm on the beam axis in a water sample, for a distance between the radiation source and the sample surface of 100 cm and a radiation beam of size 10×10  cm at the sample surface orthogonal to the sample surface. We simulated these calibration conditions for a 30×30×50  cm water sample (where the z direction is parallel to the beam axis) and the beam axis through the center of the sample and computed the dose per particle $D_{d}$ at depth d on the beam axis. By assigning a dose value of 1 cGy to this point, we calculated the number N of particles that correspond to 1 MU as $N=1\;\mathrm{cGy}/D_{d}$. The dose in Gy and the number of emitted Cherenkov photons (described below) for the IMRT and VMAT treatments were calculated by multiplying the simulated values per particle by N and by the corresponding MUs values in Table 1.

Charged particles that have an energy exceeding a threshold value of ∼0.2  MeV^31^ also induce Cherenkov light in biological tissue. The number of Cherenkov light photons (N) generated per unit length (x) and per unit wavelength is calculated by GAMOS using the Frank–Tamm formula^1^

$$\frac{d^{2}N}{dxd\lambda }=\frac{2\pi z^{2}}{137}(1-\frac{1}{\beta (E{)}^{2}n^{2}})\frac{1}{\lambda^{2}},$$ (1)

where λ is the photon wavelength, z and n are, respectively, the atomic number and refractive index of the tissue, and β is the ratio between the phase speed of the charged particle and the speed of light in a vacuum. Light transport in the tissue is simulated in GAMOS through stochastic sampling, using spectrally dependent tissue-specific optical parameters. The number of photons emerging on the patient’s surface in all directions in each voxel was interpolated across a finite element model of the patient’s surface (obtained from CT and RTStruct data^32^) to obtain a representation of the Cherenkov light distribution at the surface.

## 3 Results

### 3.1 Dose Delivery and Cherenkov Light Emission

**Fig. 3:**
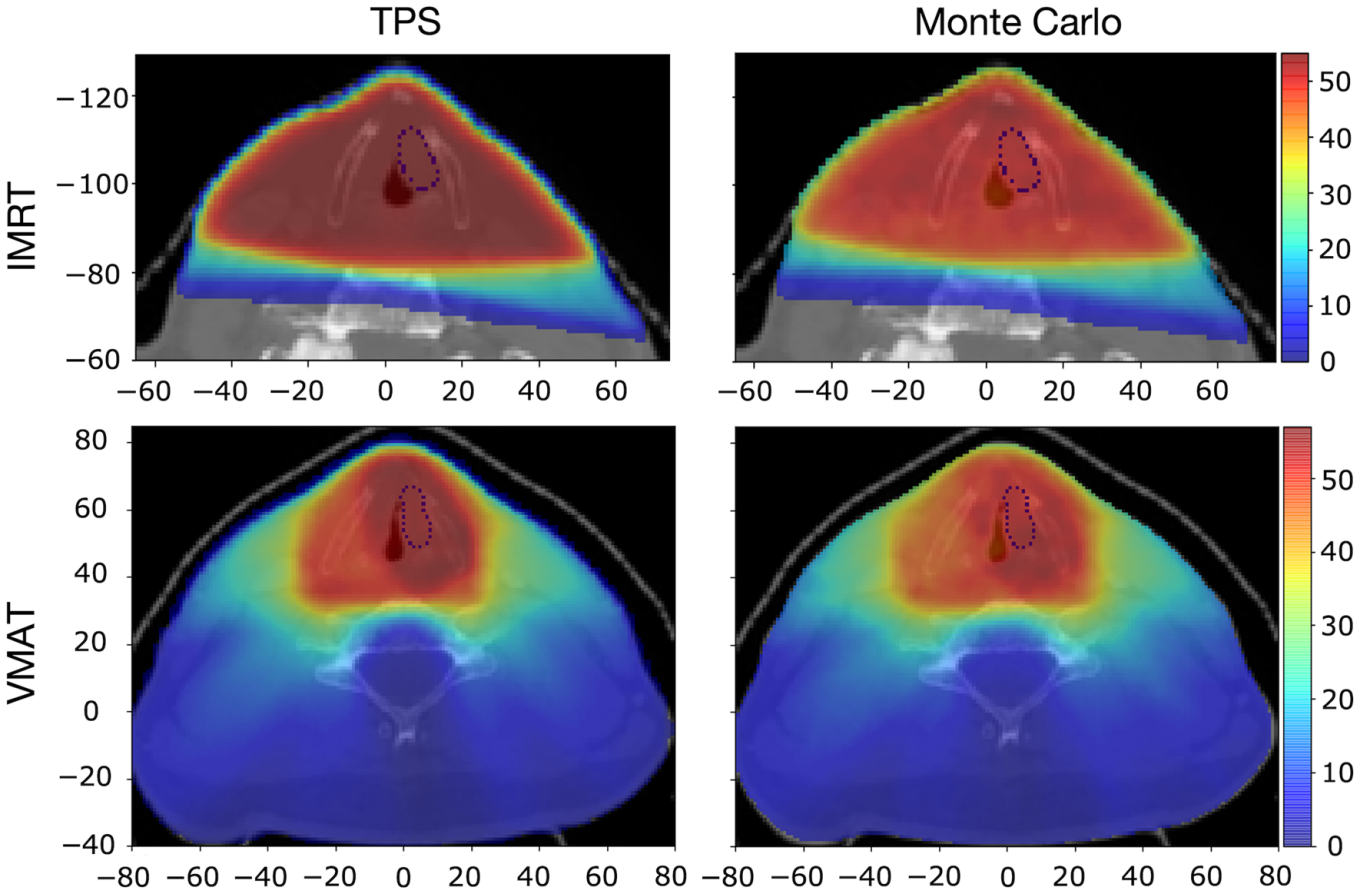
Dose distribution for IMRT and VMAT (in Gy) from the treatment planning system and Monte Carlo simulations on an axial slice at the tumor center. The tumor is indicated by a dotted contour, the axes units are in mm, and only a relevant part of the axial slice is presented for the IMRT patient.

Figure 3 presents the distribution of the total dose deposited during all treatment sessions, provided by the clinical treatment data (treatment planning system) and computed through Monte Carlo simulations. The dose distribution provided by the clinical data had a cut-off of 10% of the maximum value, and the same threshold was applied to the simulated dose distributions presented in Fig. 3. We obtain an overall good agreement among these distributions, with a relative agreement of 94% and 98% in terms of the total dose in the tumor for IMRT and VMAT, respectively. This indicates a successful simulation of the delivered dose. In what follows, we present various quantities computed for one single treatment session, which is relevant for practical applications.

**Fig. 4:**
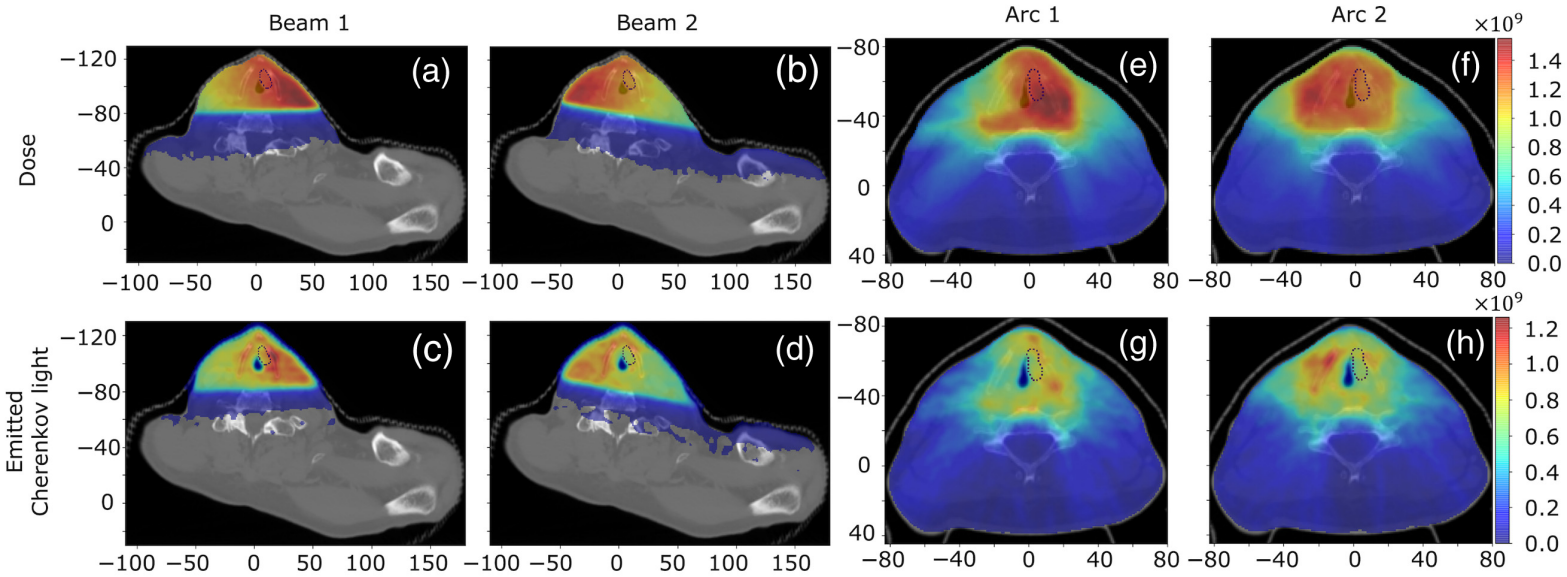
Distribution of delivered dose (in Gy) and the emitted Cherenkov light (photons per ${\mathrm{mm}}^{3}$) for each of the IMRT treatment beams [(a)–(d)] and VMAT treatment arcs [(e)–(h)] in an axial slice across the center of the tumor. The tumor is indicated by a dotted contour, and axes units are in mm.

Figure 4 presents the distributions of dose and Cherenkov light emitted in the tissue (in the 500  to 1200 nm range) for the IMRT and VMAT treatments. A cut-off of 1% of the maximum value has been applied to the distributions presented here. The distribution of Cherenkov light overlaps with that of high dose, and these distributions vary across both treatments and each treatment beam or arc due to the different beam characteristics such as incidence angle and intensity and spatial modulation. In particular, for the patient used in the IMRT study, the radiation beam is delivered laterally to the neck, and dose is deposited and Cherenkov light is produced just on the front part of the neck. For the VMAT treatment, the radiation is delivered from around the front of the patient, and some dose is delivered and some Cherenkov light is emitted also toward the back of the neck. We also note differences between the distribution of emitted Cherenkov light and deposited dose. These are associated with the tissue type–dependent threshold energy of charged particles to induce Cherenkov light and the stronger dependence of Cherenkov light emission on the density of various tissue constituents [through $z^{2}$ and n as expressed in Eq. (1)] than the deposited dose (which only depends linearly on z^33^).

### 3.2 Cherenkov Light on the Patient’s Surface

**Fig. 5:**
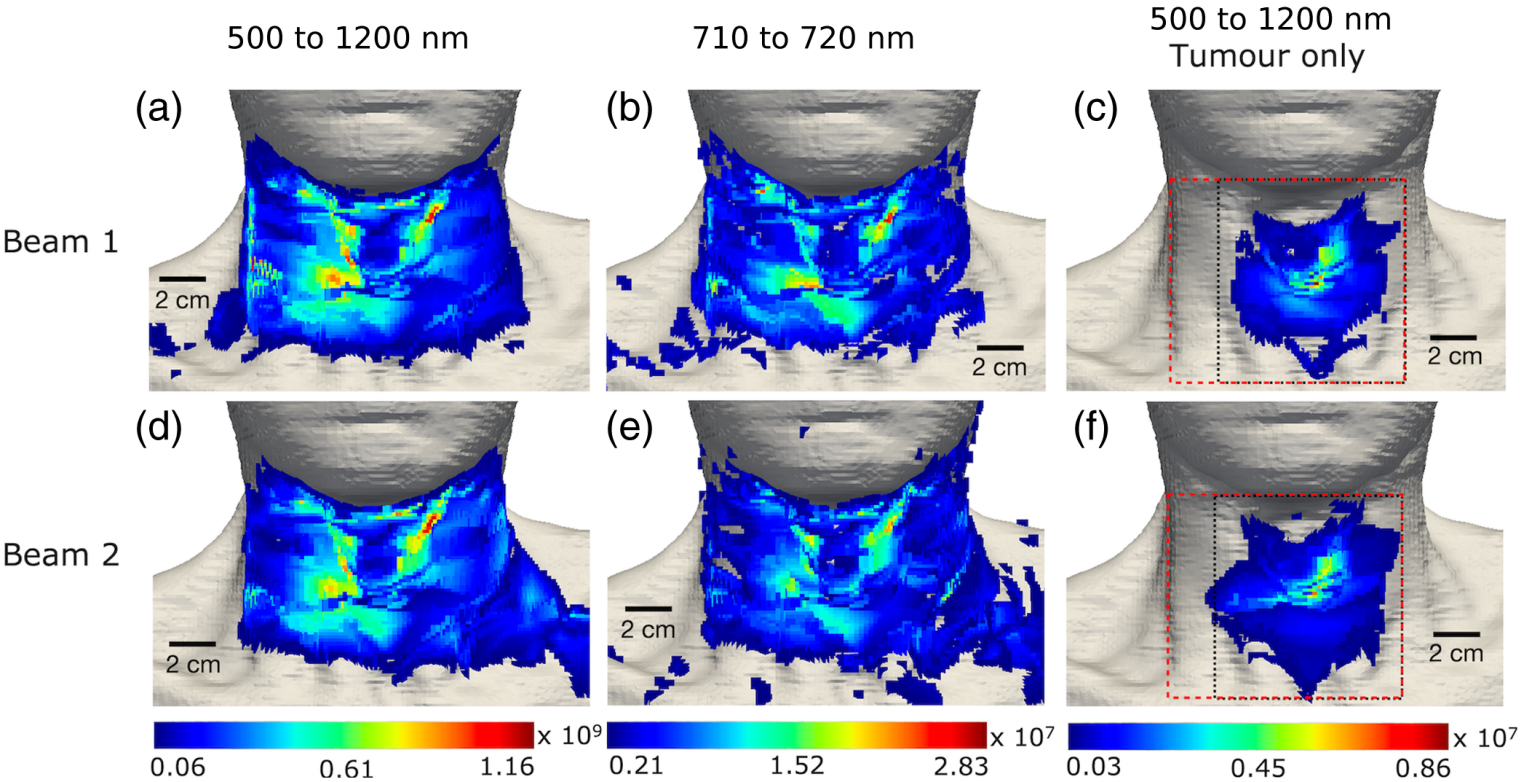
IMRT treatment. The Cherenkov light distribution at the patient’s surface for each IMRT beam, corresponding to photons emitted everywhere in the patient in the 500 to 1200 nm spectral range [(a) and (d)], in the 710 to 720 nm spectral range [(b) and (e)], and emitted only in the tumor in the 500 to 1200 nm spectral range [(c) and (f)]. The reduced measurement areas 1 and 2 are represented by a dashed red and dotted black box, respectively, in the right column.

**Fig. 6:**
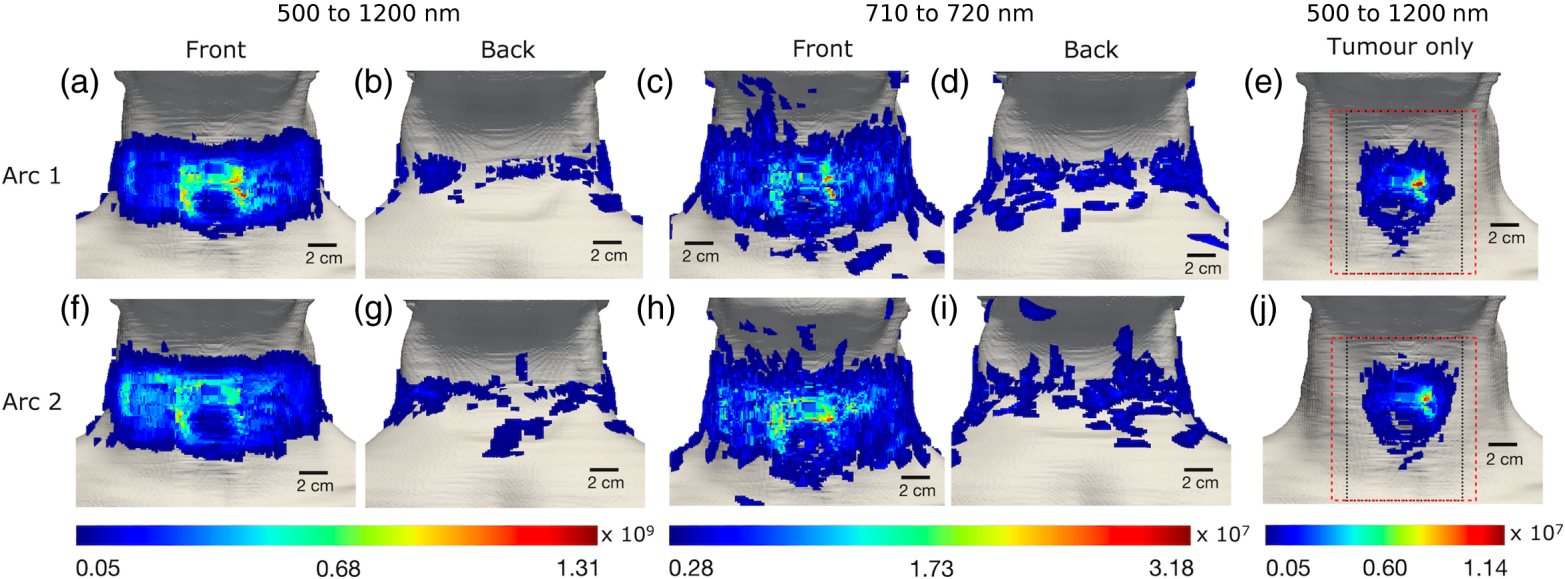
VMAT treatment. The Cherenkov light distribution at the patient’s surface for each VMAT arc, corresponding to photons emitted everywhere in the patient in the 500 to 1200 nm spectral range [(a), (b), (f), and (g)] in the 710 to 720 nm spectral range [(c), (d), (h), and (i)], and emitted only in the tumor in the 500 to 1200 nm spectral range [(e) and (j)]. The reduced measurement areas 1 and 2 are represented here by a dashed red and dotted black box, respectively, in the right column.

Figures 5 and 6 present the spatial distribution of the spectrally integrated Cherenkov light intensity at the patient’s surface for the IMRT and VMAT treatments, respectively. Specifically, we present the distribution of light corresponding to all photons emitted in the patient that reach the patient’s surface (for the spectral ranges 500 to 1200 nm and 710 to 720 nm), and the distribution of surface light that was emitted in the tumor only (in the 500 to 1200 nm spectral range). The narrow spectral range of 710 to 720 nm is considered to simulate detection at discrete wavelengths, commonly used in diffuse optical tomography and spectroscopy with external light sources to probe tissue chromophores such as oxygenated and de-oxygenated hemoglobin.^11^^,^^34^ The surface light distribution is dependent on the radiation (treatment) delivery type and is concentrated on the side of the patient that is more irradiated, as also observed experimentally for other treatments.^4^ In particular, for the IMRT treatment, photons emerge on the surface only at the front of the patient (and for this reason a view from the back of the patient is not shown in Fig. 5). For the VMAT treatment, there is light emerging also on the side and at the back of the patient, although this is of much lower intensity compared with that emerging at the front of the patient. To obtain these surface distributions, a median filter was applied to simulated data as well as thresholding to only consider values above 5% and 10% of the maximum value for the distributions for 500 to 1200 nm and 710 to 720 nm, respectively. This resulted in filtering some of the near-infrared light (of lower intensity) in the distribution for the 500 to 1200 nm spectral range, which together with the increased noise in the data for the smaller sampling wavelength range of 710 to 720 nm explains the difference in the distributions for the two spectral ranges. From Figs. 5 and 6, it can be seen that the light emitted in the tumor emerges on the patient’s surface on a relatively well-defined region, herein referred to as the tumor spot, which is an area of high intensity and is similar to both IMRT beams and both VMAT arcs, respectively. This suggests that measurements of surface light over smaller areas such as those illustrated in Figs. 5 and 6 can still enable probing the tumor.

**Fig. 7:**
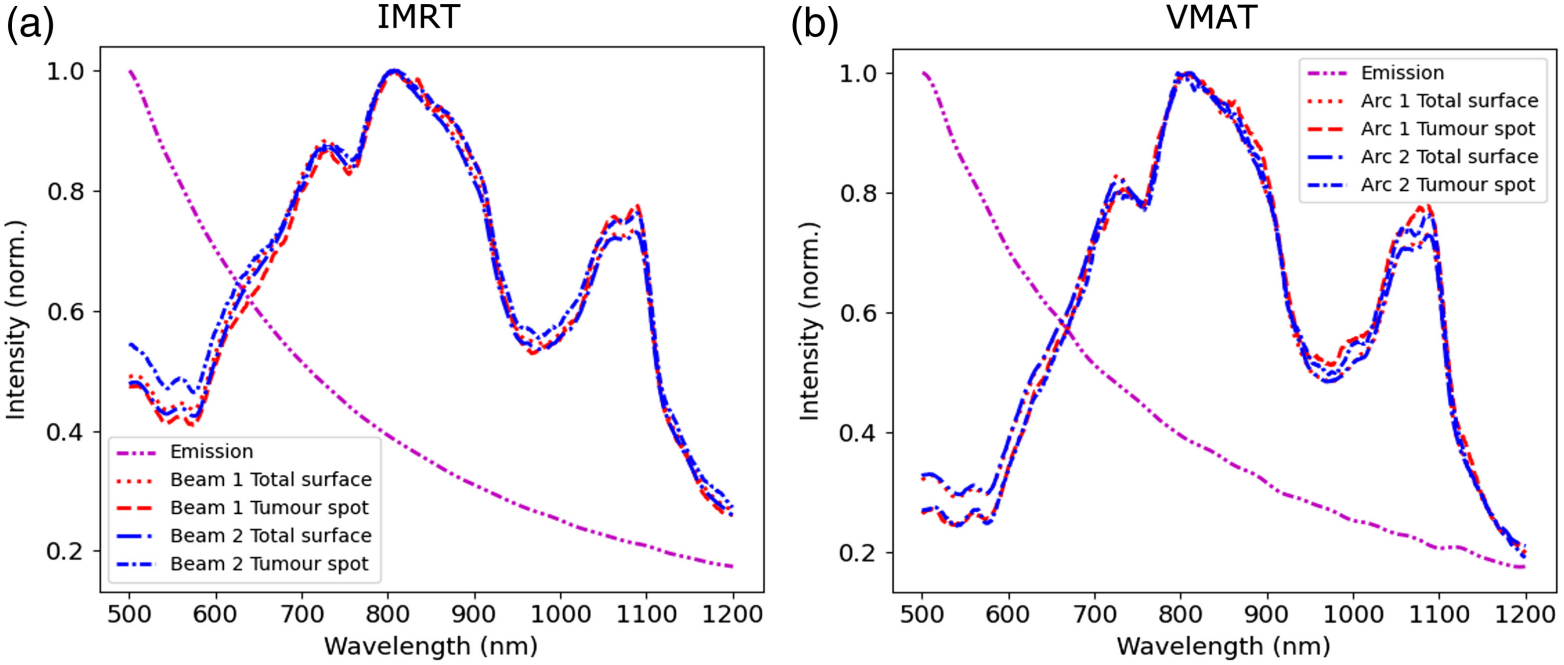
Normalized spectra of Cherenkov light spatially integrated over the entire patient volume (for the emitted light), and over the total surface of the patient and the tumor spot (for light at the surface) for each IMRT beam (a) and VMAT arc (b).

In Fig. 7, we present the spectrum of spatially integrated Cherenkov light emitted in the tumor and of spatially integrated light emerging on the entire patient’s surface and on the tumor spot (that corresponds to photons emitted at various depths within the patient). Although the intensity of the emitted Cherenkov light is higher at lower wavelengths λ and presents a $1/\lambda^{2}$ dependency,^1^ the surface light is predominant in the near-infrared region and presents two spectral maxima resulting from the spectral characteristics of the tissue optical attenuation. In addition, although the spectrum of surface light is similar for the IMRT and VMAT treatments, larger intensity values in the 500 to 650 nm range are observed for the IMRT treatment. This difference is related to how the tissue is irradiated. In the 500 to 650 nm spectral range, tissue optical absorption is large and the surface Cherenkov light originates near the surface. In the IMRT treatment, each radiation beam delivers nearly half of the prescribed dose. This results in a stronger irradiation of the superficial tissue in front of the patient and hence more Cherenkov light emission in this region. In the VMAT treatment, the radiation is delivered across a large gantry angle range, and the beam intensity for each gantry position is reduced relative to the IMRT beam intensity, reducing the irradiation of the superficial tissue.

### 3.3 Origin of the Surface Light

**Fig. 8:**
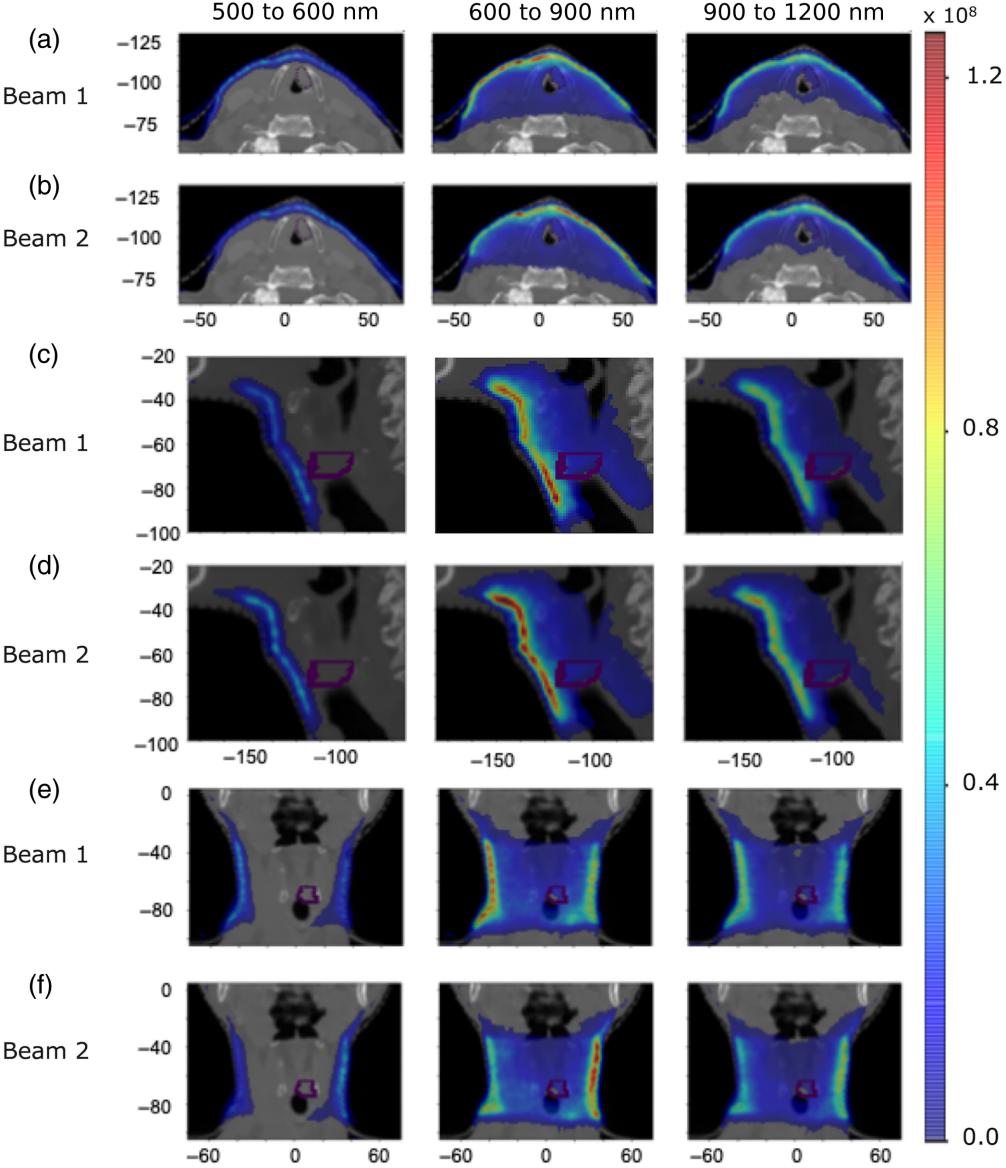
IMRT Treatment. The distribution of emitted Cherenkov light (photons per ${\mathrm{mm}}^{3}$) that contributes to the light across the whole patient’s surface within three spectral ranges for each radiation beam, in axial [(a) and (b)], sagittal [(c) and (d)], and coronal [(e) and (f)] slices at the tumor center. The tumor is indicated by a dotted contour and the axes units are in mm.

**Fig. 9:**
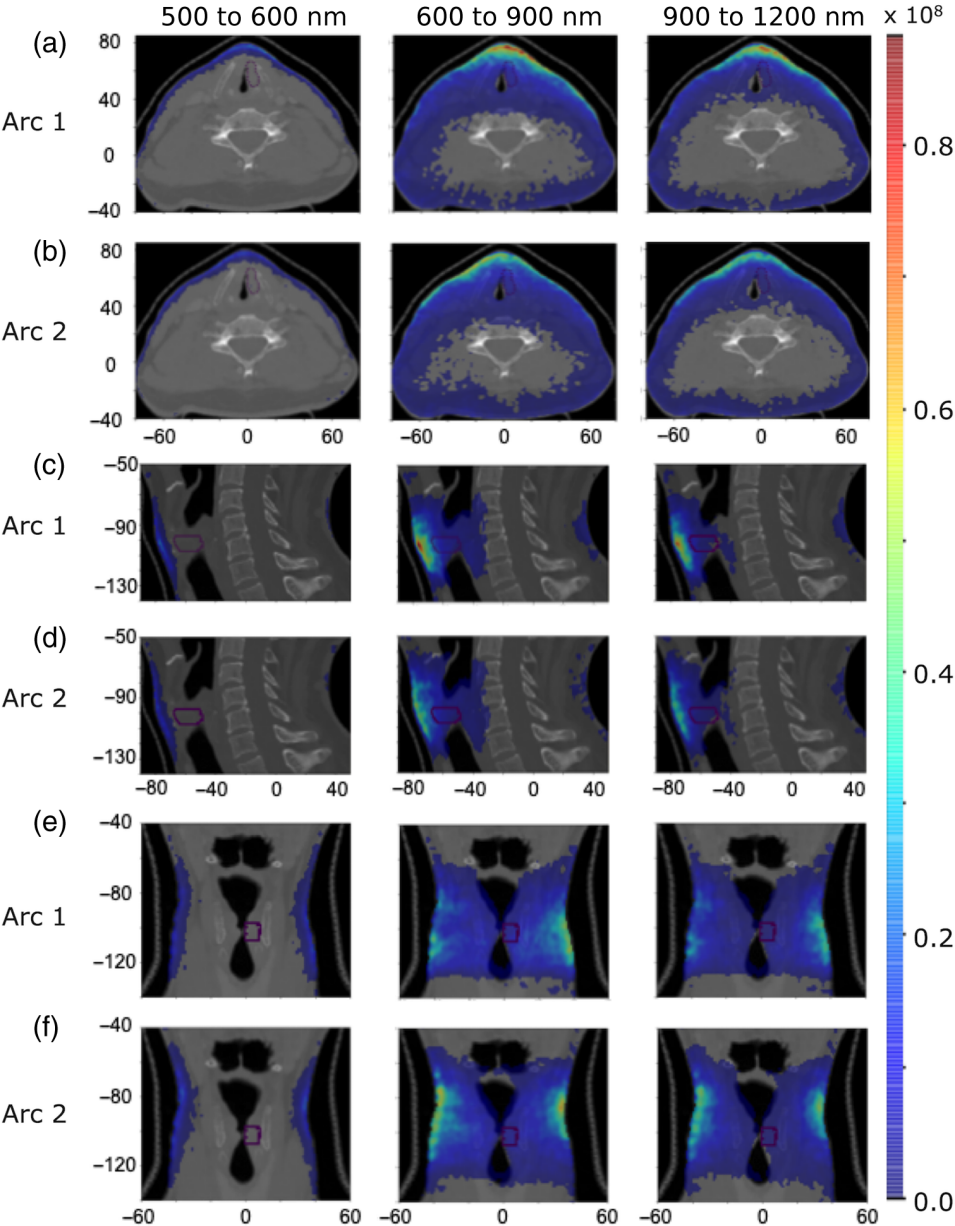
Same as for Fig. 8 but for VMAT treatment.

**Fig. 10:**
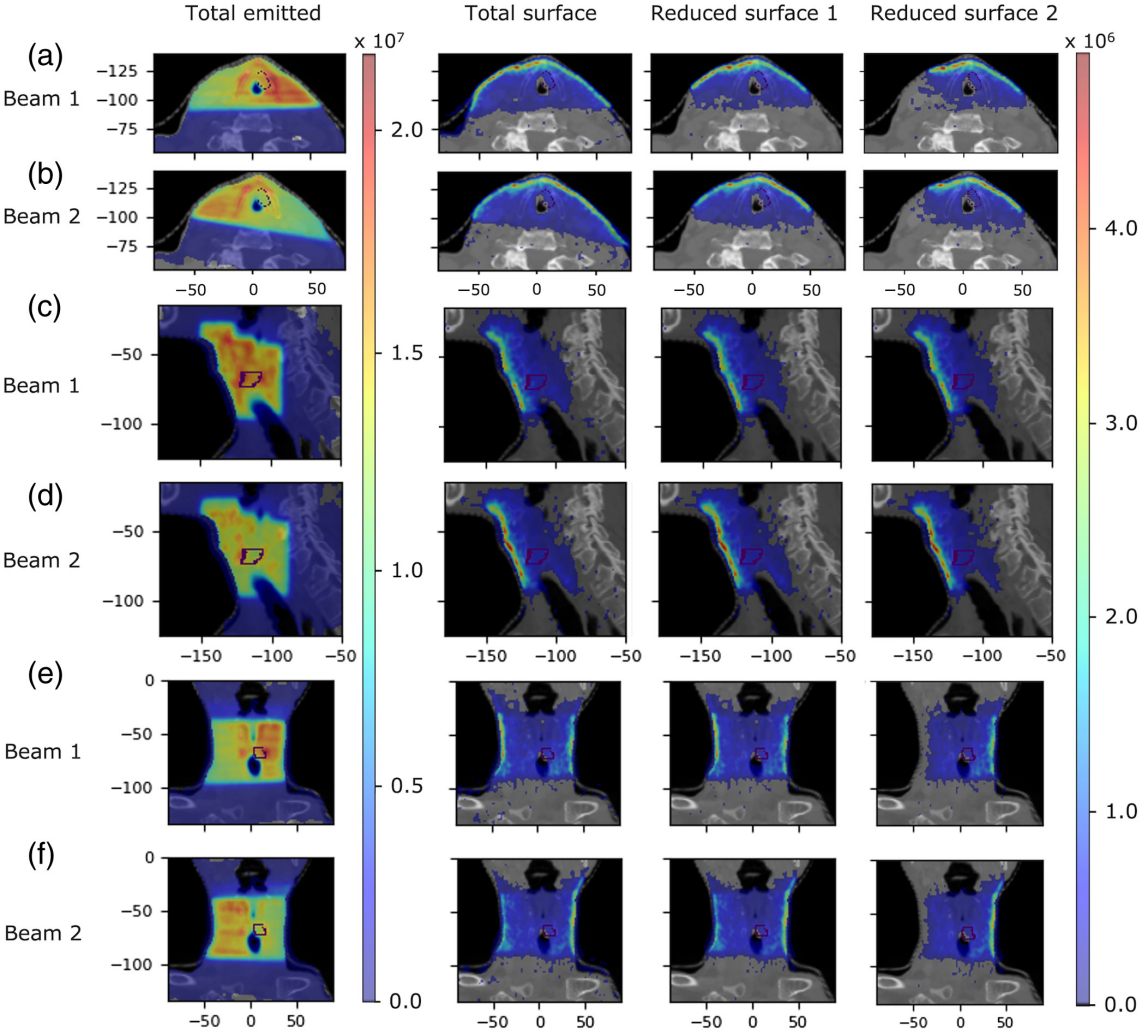
IMRT Treatment. The distribution (photons per ${\mathrm{mm}}^{3}$) for the 710 to 720 nm spectral range of the total emitted Cherenkov light and of the emitted light that contributes to the surface light across the entire patient’s surface and across the surfaces 1 (of width 10 cm) and 2 (of width 8 cm) presented in Fig. 5 for each treatment beam, in axial [(a)  and (b)], sagital [(c) and (d)], and coronal [(e) and (f)] slices at the tumor center. The tumor is indicated by a dotted contour and the axes units are in mm.

**Fig. 11:**
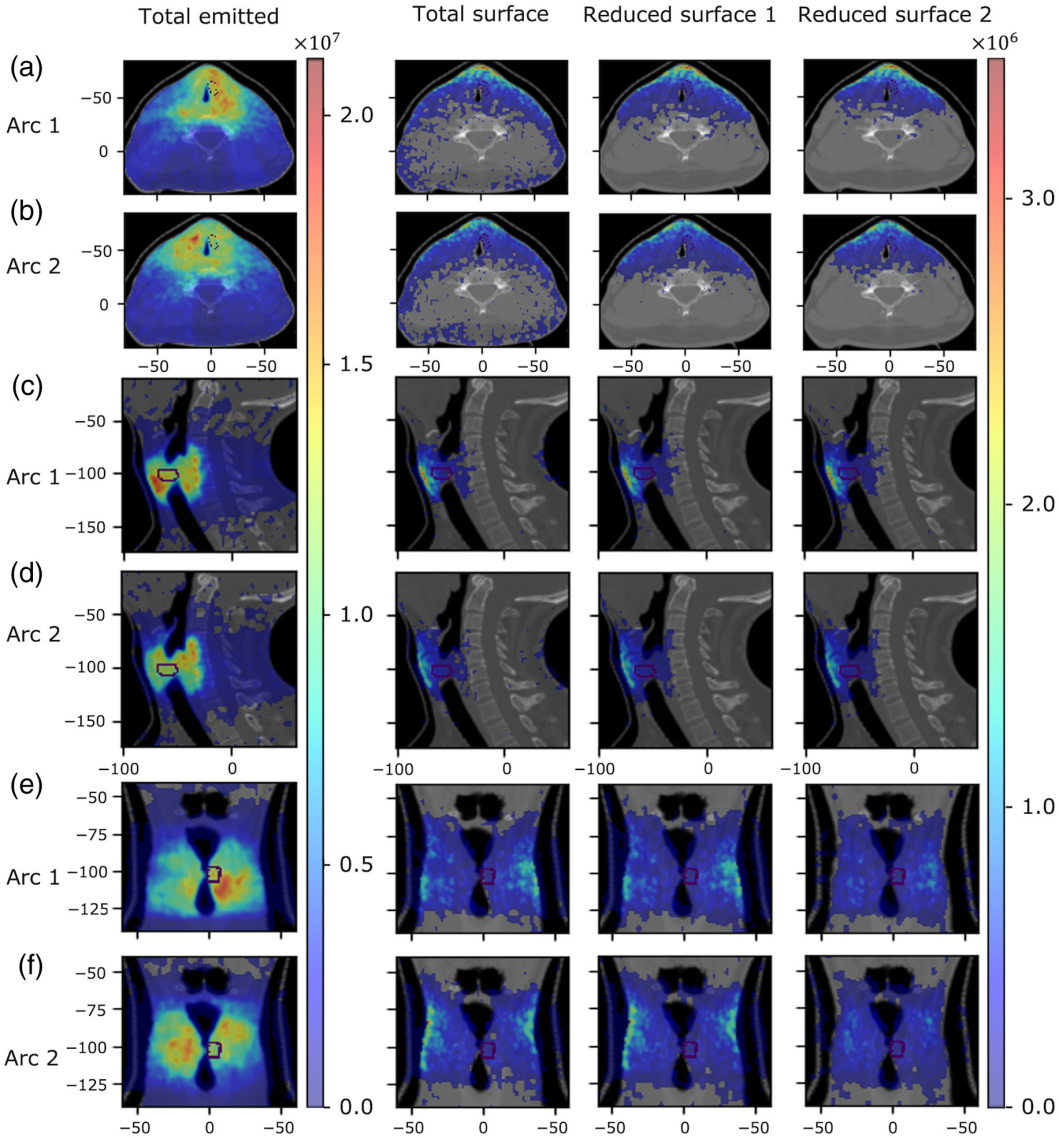
Same as for Fig. 10 but for each VMAT treatment arc and for the reduced measurement areas 1 (of width 10 cm) and 2 (of width 8 cm) illustrated in Fig. 6, in axial [(a) and (b)], sagittal [(c) and (d)], and coronal [(e) and (f)] slices at the tumor center.

We now investigate the origin of Cherenkov light within the patient that emerges on certain regions on the patient’s surface. Figures 8 and 9 present the spatial distribution of the emitted light contributing to the surface light across the entire patient’s surface for the IMRT and VMAT, respectively, for three broader spectral ranges selected based on the spectrum of surface light. Figures 10 and 11 present, for the spectral range 710 to 720 nm, the spatial distribution of the total emitted light and the emitted light that contributes to the surface light across the entire patient’s surface and across the two reduced areas (that include the tumor spot), shown in Figs. 5 and 6, respectively, of widths 10 and 8 cm. These results show that above 600 nm, light emitted deeper in tissue and in the tumor could in principle reach the patient’s surface, and measurements of surface light in this spectral region could enable probing of the tumor. The depth at which the tissue can be probed is the same for both IMRT beams and both VMAT arcs, and reducing the measurement area does not affect the ability to probe the tumor. In addition, these results together with those in Figs. 5 and 6, showing that the distribution of the emitted light that contributes to the surface light across the entire patient’s surface is similar to that of the emitted light that contributes to surface light across the surface 1, indicate that restricting the measurements of surface light to an area of ∼10  cm width suitably positioned is not expected to result in a significant level of data incompleteness and errors for tomographic image reconstruction.

## 4 Discussion and Conclusion

Numerical experiments with clinical patient data were performed to investigate the characteristics of Cherenkov light emitted within the patient during IMRT and VMAT treatments of laryngeal cancer and its potential for probing the tumor. This study demonstrates that Cherenkov light emission is localized in regions of high dose and is predominantly in the ultraviolet spectral region. Light emerging at the patient’s surface is mostly distributed on the front of the patient and is predominantly in the near-infrared spectral range. It originates deep enough in the tissue and could potentially be used to probe the tumor during the treatment course. Cherenkov light emitted within the tumor and immediately surrounding tissue emerges at the patient’s surface on a high-intensity well-defined region, referred to here as tumor spot, which is roughly the same for all radiation beams or arcs of the specific treatments.

In practice, the distribution and intensity of emitted Cherenkov light (internal light sources) as well as the tumor spot can be determined (via simulations) prior to treatment based on readily available clinical data, as presented in this study. Then, the knowledge of the internal sources can be combined with surface light measurements to determine the optical properties of the tissue. This, however, requires image reconstruction algorithms based on internal (rather than external) light sources, possibly combining approaches such as those used in diffuse optical tomography,^11^ tomographic imaging with an internal source,^35^ bio-luminescence tomography,^36^ or AI techniques for optical imaging^37^ while also exploiting the wealth of prior imaging available in radiation therapy. This study illustrates the spatial characteristics of the internal sources and of the light at the patient’s surface and provides insight for developing such image reconstruction algorithms.

This study suggests that Cherenkov light measured on reduced areas on the patient’s surface that contain the tumor spot could probe the tumor. Restricting the light measurements to smaller areas could enable easier integration of a Cherenkov light–based tomographic imaging technology with the radiotherapy system. In this case, image reconstruction would be performed using the total light emitted in the tissue as internal sources but reduced (incomplete) surface measurements, rather than measurements of all the surface light having as the source the total emitted light. Although image reconstruction based on incomplete datasets would still be possible, data incompleteness affects the quality of the reconstructed image, and usually approaches that depend on the severity of the effect of data incompleteness are needed to tackle this problem. This study shows that the distribution of the emitted light within the patient that contributes to light on some of reduced areas on the patient’s surface (specifically, the larger regions, positioned as illustrated in Figs. 5 and 6) is similar to that of the light that emerges across the entire patient’s surface, indicating that the effect of data incompleteness on image reconstruction based on reduced measurements is expected to be not too strong. In practice, such reduced measurement regions are patient- and treatment type–specific and would necessarily need to include the tumor spots. Further refinement of these regions can be done by balancing their size and location with the difference (ideally small, to avoid large errors in image reconstruction) between the distribution within the tissue of the emitted light that emerges to these areas and the distribution of the emitted light that emerges on the whole patient’s surface. Determining the tumor spot and a measurement area refinement study can be done prior to the treatment as done for Figs. 5 and 6, and Figs. 8 and 9, respectively, using the patient RT and CT data, known tissue optical characteristics, and available validated simulation software.^22^^,^^23^

Important developments have been made recently in terms of the sensitivity of Cherenkov light measurements in radiation therapy, in particular, for applications to dosimetry.^3^^,^^4^^,^^38^^–^^40^ By quantifying the light at the patient’s surface (for the patient anatomies and the values of the tissue optical parameters considered), this study indicates the level of detection sensitivity needed in the case of radiation therapy of the larynx, thus providing additional insight for developments. In addition, the quantitative information about the emitted Cerenkov light in the body provides insight for further developments in radiation dosimetry and tissue imaging with exogenous contrast agents.^19^

## Data Availability

Data presented herein are available in figshare at https://figshare.com/articles/dataset/Figure_data/29150357.
